# Knowledge, attitude and practices on cervical cancer screening among undergraduate female students in University of Gondar, Northwest Ethiopia: an institution based cross sectional study

**DOI:** 10.1186/s12889-021-10853-2

**Published:** 2021-04-23

**Authors:** Alem Getaneh, Birhanemeskel Tegene, Teshome Belachew

**Affiliations:** 1 Department of Medical Microbiology, School of Biomedical and Laboratory Science, College of Medicine and Health Sciences, University of Gondar, 196 Gondar, Ethiopia; 2 St. Paul’s Hospital Millennium Medical College, Addis Ababa, Ethiopia

**Keywords:** KAP, Cervical cancer, Undergraduate female university students, Ethiopia

## Abstract

**Background:**

Cervical cancer is a major public health problem. In the world, cervical cancer is the fourth most common cancer among women and it is one of the leading causes of cancer mortality in females. It is the second most common women cancer in Ethiopia with almost 6300 new cases and 4884 deaths annually. Despite the high burden of new cases and deaths, there is a scarcity of data on knowledge, attitude and practices (KAP) towards cervical cancer screening among female university students in Ethiopia particularly in the study area. Therefore, the present study was aimed to assess the KAP of undergraduate female students towards cervical cancer screening.

**Methods:**

An institution based cross-sectional study was conducted in April 2018 at the University of Gondar, College of Medicine and Health Sciences undergraduate female students. Pretested, self-administered questionnaire was used for data collection. Four hundred and three female students were recruited by a simple random sampling method and the data were entered and analyzed using SPSS version 20 statistical packages. Descriptive data analysis was used to report the results.

**Results:**

More than half of the respondents (59.3.3%) had good knowledge, whereas nearly 67.7% of the respondents had favorable attitude towards cervical cancer. However, less than 1% of the respondents had been screened for cervical cancer.

**Conclusion:**

Although undergraduate female students had apparently good knowledge and favorable attitude, their practices on cervical cancer screening were quite low. Therefore, the health sectors and the gender streaming office of the university mobilize students to strengthen the uptake the cervical cancer screening practice.

## Background

Cervical cancer is cancer of uterine cervix that causes a serious public health problem [1]. It is a major cause of morbidity and mortality among women’s in the world [2]. The main cause for cervical cancer is the Human Papilloma Virus (HPV) which is transmitted through sexual intercourse. Human Papilloma Virus is a group of viruses that commonly infect the reproductive tract of sexually active young adult men and women, Today, more than 150 different HPV types have been identified, Particularly the age of 22–25 years [3]. Among these, thirteen HPV types such as HPV16, 18, 31, 33, 35, 39, 45, 51, 52, 56, 58, 59, and 68 are identified as “high risk HPV” (HR-HPV) due to their relatively high carcinogenic potential leading to the development of cervical cancer among more than 150 HPV strains being found [4]. HPV types 16 and 18 are responsible for about 70% of all cervical cancer cases worldwide. Human Papilloma Virus vaccines that prevent HPV 16 and 18 infections are now available and have the potential to reduce the incidence of cervical and other anogenital cancers [5]. Early-onset of sexual activities, multiple sexual partners, extended use of oral contraceptives, immune-suppression and smoking are other known risk factors [6].

Malignant cervical cancer is characterized by vaginal bleeding between menstrual periods, pelvic pain, vaginal bleeding during/after sexual intercourse and vaginal secretion. However, it is one of the most easily preventable forms of female cancer. A key aspect of its prevention is the identification of the premalignant form by cervical screening [7–9] and HPV vaccination [2]. However, there are so many reasons mentioned as barriers towards cervical cancer screening in developing countries such shortage and underutilization of screening facilities, lack of awareness and poor attitude towards cervical cancer and risk factors, beliefs about cervical cancer, feeling healthy, stigma, fear of the test results and fear of marital disturbance [10, 11].

Cervical cancer is the fourth most common cancer among women worldwide, ranking after breast cancer, colorectal cancer and lung cancer. Approximately 570, 000 cases of cervical cancer and 311, 000 deaths from the disease occurred in 2018. More than 85% of the estimated deaths occur in low and middle income countries [11, 12]. Cervical cancer is the second most common cancer after breast cancer among women in the developing world and is responsible for the deaths of 230,200 and the new cases of 444,500 annually [13] The rate of cervical cancer was five times higher in developing countries (25 per 100,000) compared with more developed countries (5 per 100,000) [14].

In Ethiopia, cervical cancer is the second most common cancer and the second most deadly cancer among Ethiopian women with almost 6300 new cases and 4884 deaths annually [15].

Despite the growing number of cervical cancer cases in Ethiopia, there is still a gap in knowledge, attitude and practice towards cervical cancer screening. No previous study was done on the KAP towards cervical cancer and its screening among the University of Gondar, College of Medicine, and Health Sciences undergraduate female students. Therefore, the present study was aimed to assess their baseline KAP towards cervical cancer and its screening.

## Methods

### Study area

The study was conducted at the University of Gondar, College of Medicine and Health Sciences in Gondar town, northwest Ethiopia. Gondar town is located in northwestern Ethiopia at a latitude of 12“36’North, and longitude of 37”28’East. The University of Gondar is located in the west of the Central Gondar Administrative Zone, which is, 747 km far from Addis Ababa in the Northwest direction [16]. According to the central and statistical agency of Ethiopia report in 2015, the town has 12 sub city, 22 urban and 11 rural kebeles with a total projected population of 323,900. In the town; there is one university which has 5 campuses. Of these College of Medicine and Health Sciences is the one that provides a service for more than 5400 students every year. The college has one institution, 5 schools and 12 departments. In the year 2018, the total number of female students was 1746.

### Study design and period

An institution-based cross-sectional study was conducted at the University of Gondar, College of Medicine, and Health Sciences on April 2018.

### Population

#### Source population

All female students in College of Medicine and Health Sciences were used as sources of population.

#### Study population

All selected regular undergraduate female students in College of Medicine and Health Sciences were used as a study population.

#### Eligibility criteria

All regular Medicine and Health Sciences under graduate female students were included in the study.

### Operational definition

#### Knowledge

Participants who have scored greater than or equal to 6 correct answers from 11 knowledge questions were considered to have good knowledge, and those who had scored less than 6 answers were considered to have poor knowledge.

#### Attitude

Participants who have scored greater than or equal to 5 correct response from 9 attitude questions were considered to have a favorable attitude and those who had scored less than 5 were considered to have unfavorable attitude.

#### Screening practice

Participants who are screened for cervical cancer at least once.

### Sample size determination

The required sample size was determined by using the single population proportion formula based on the following assumptions. The proportion of knowledge/attitude was taken from the results of a study done in Mizan Tepi University, Ethiopia, 61% [17], with 95% CI, α-level 5%, and considered the margin of error 5%. The minimum sample size was calculated based on the following formula:

$$ \mathrm{n}={\left(\mathrm{Za}/{}_2\right)}^2\ \mathrm{P}\ \left(1-\mathrm{P}\right)/{\mathrm{d}}^2;\mathrm{n}={(1.96)}^2\ast 0.61\left(1-0.61\right)/{(0.05)}^2=367 $$

Where: n = sample size.

Z α /_2_ = normal distribution value at 95% CI (Z = 1.96).

P = the successful proportion of knowledge/attitude (0.61).

d = margin of error (5%).

Therefore, the final sample size after adding 10% non-response rate was 403female students.

### Sampling technique

The study population was stratified based on the department of their discipline. Participants were selected from 12 departments of the University of Gondar, College of Medicine and Health Sciences by using simple random sampling technique. The sample size was proportionally allocated for each department based on their class size by using the formula: ni = n*Ni/N.

Where n = the total sample size (403); ni = required sample size in each department, Ni = Number of female students in each department; N = Total number of female students in the study area (1746).

### Data collection

The data was collected by using a self-administrated questionnaire that contains different items like socio-demographics, knowledge, and attitude and practice questions towards cervical cancer screening. Questionnaires were adapted from different pieces of literature from previous studies [17, 18]. These questions explored the respondent’s knowledge, attitude, and practices about cervical cancer screening. The questionnaires were translated from English to Amharic for data collection and vice-versa for data entry for ensuring its consistency.

### Data quality management

A pre-test was done on 5% of the questionnaire to make sure the questionnaire was appropriately structured and to ensure its consistency. Two days of training were given to data collection facilitators for improving the data collection process. Close supervision was done by the principal investigator for ensuring the completeness of questionnaires at the time of data collection. Finally, data editing and clearance were done for the proper management of data.

### Data management and analysis

Data were entered after encoded the completed questionnaires and analyzed using SPSS version 20 statistical packages. Descriptive data analysis was used to describe the knowledge, attitude, and practices for cervical cancer screening. The results were presented in mean, standard deviation, texts and tables.

## Results

### Socio-demographic characteristics of participants

A total of 403 undergraduate female health science students were included in this study. The overall response rate was 100%. The minimum age of the study participants was18 and the maximum age was 25 with the mean (±SD) age of 21(±1.5) years. Among these, the majority 217 (54%) of the respondents were between the age range 21–25 years while the remaining 186 (46%) were between the age range 18–20 years. About 322(79.9%) of the respondents were orthodox Christian and 256(63.5%) were Amhara in ethnicity. Regarding marital status, 390(96.8%) of the respondents were single followed by 11(2.7%) married, 1 (0.2%) divorced, and 1 (0.2%) separated. About 333 (82.6%) of the respondents, came from urban parts of Ethiopia.

### Knowledge of the respondents towards cervical cancer

From this study, more than half of the respondents, 239 (59.3%) had good knowledge, whereas the remaining two-fifth 164(40.7%) of respondents characterized by poor knowledge. The majority 363 (90%) of the respondents, heard about cervical cancer and its screening from different information sources. Nearly one-third (32.8%) of the respondents who heard about cervical cancer and its screening, got the information from their teachers. Less than one-third, 142 (35.2%) of the respondents knew about the causative agent of cervical cancer. More than half of the respondents, 211(52%) knew about the sign and symptoms of cervical cancer, whereas 192(47.6%) of the respondents didn’t know. Among respondents who knew the sign and symptoms, 84 (20.8%) of respondents said that Vaginal foul-smelling discharge is the symptom of the disease whereas, 83(20.6%) of the respondents reported that irregular vaginal bleeding is the symptom of the disease. However, one-third, 133 (33%) of participants had no idea what factors raise the chance of getting cervical cancer, whereas one-quarter of participants 101 (25%) reported that having multiple sexual partners is a risk factor for the disease. Most of the respondents, 296 (73.4%), knew as early treatment can cure cervical cancer. Regarding knowledge on vulnerability for the Pap smear test, 195 (48.4%) of the respondents pointed out that all women > 25 years should get the Pap smear test (Table 1).

**Table 1:** Knowledge towards cervical cancer and its screening at the University of Gondar, Ethiopia, April, 2018 (*n* = 403)

Variables	Categories	Number	Percent (%)
Have you ever heard about cervical cancer?	Yes	363	90.1
	No	40	9.9
Where did you learn about cervical cancer?	Teachers	119	32.8
	News media	108	29.8
	Health institutions	98	27
	Family, friends, neighbors	30	8.3
	Magazine	8	2.2
What is causative agent of cervical cancer?	Virus	142	35.2
	Bacteria	54	13.4
	Fungi	20	5
	Parasite	16	4
	Don’t know	171	42.4
What are the symptoms of carcinoma of the cervix?	Vaginal foul smelling discharge	84	20.8
	Vaginal irregular bleeding	83	20.6
	Post coital bleeding	15	3.7
	All of the three symptoms	29	7.2
	Don’t know	192	47.6
Do you know the Risk factors for cancer of the cervix?	Having multiple sexual partners	101	25.1
	Human papilloma virus	63	15.6
	Early sexual intercourse	48	11.9
	Cigarette smoking	11	2.7
	All the four risks	47	11.7
	Don’t know	133	33
How can a person prevent getting cancer of the cervix?	Avoid multiple sexual partners	101	25.1
	Avoid early sexual intercourse	68	16.9
	HPV vaccination	33	8.2
	Quit cigarette smoking	19	4.7
	All the four prevention mechanisms	42	10.4
	Don’t know	140	34.7
Can cancer of the cervix be cured in its earliest stages?	Yes	296	73.4
	No	19	4.7
	Don’t know	88	21.8
How can someone with cancer of the cervix be treated?	Surgery	94	23.3
	Specific drugs are given by a hospital	92	22.8
	Radiotherapy	92	22.8
	All	19	4.7
	Don’t know	106	26.3
Screening frequency	Once a year	240	59.6
	Every 3 years	83	20.6
	Every 5 years	30	7.4
	All	5	1.2
	Don’t know	45	11.2
Who should be screened?	Women of > 25 years	195	48.4
	Prostitutes	83	20.6
	Elderly women	30	7.4
	All	51	12.7
	Don’t know	44	10.9
Which screening method do you know?	Papanicolau smear	152	37.7
	Biopsy	108	26.8
	Visual inspection with acetic acid	39	9.7
	All	35	8.7
	Don’t know	69	17.1
**Compute Knowledge of respondents**	Good	239	59.3
	Poor	164	40.7

### Attitude towards cervical cancer

More than two-thirds of the respondents 273(67.7%) had a favorable attitude, whereas the remaining respondents 130 (32.3%) had unfavorable attitude towards cervical cancer and its screening. The majority of the respondents, 361(89.6%), had favorable attitude towards early detection. Only 229 (56.8%) of the respondents responded that they have the chance of acquiring cervical cancer. More than one-quarter of the respondents 110 (27.3%) thought that cervical carcinoma is transmitted from person to person. The majority of the respondents 348 (86.4%) thought that screening helps to prevent cervical cancer (Table 2).

**Table 2:** Attitudes towards cervical cancer at the University of Gondar, northwest Ethiopia, April, 2018 (*n* = 403)

Variables	Categories	Number	Percent (%)
Do you think it is helpful to detect cervical cancer early?	Strongly agree	224	55.6
	Agree	137	34
	Neutral	32	7.9
	Disagree	9	2.2
	Strongly disagree	1	0.2
Do you believe that you have the chance of getting Cervical Cancer?	Strongly agree	81	20.1
	Agree	148	36.7
	Neutral	48	11.9
	Disagree	106	26.3
	Strongly disagree	20	5.0
Do you believe that getting Cervical Cancer is a serious for you?	Strongly agree	235	58.3
	Agree	138	34.2
	Neutral	16	4.0
	Disagree	13	3.2
	Strongly disagree	1	0.2
Do you think that there are effective methods to reduce the risk of seriousness of cervical cancer?	Strongly agree	150	37.2
	Agree	204	50.6
	Neutral	33	8.2
	Disagree	10	2.5
	Strongly disagree	6	1.5
Do you think Carcinoma of the cervix is the cause of death?	Strongly agree	157	39.0
	Agree	157	39.0
	Neutral	50	12.4
	Disagree	34	8.4
	Strongly disagree	5	1.2
Do you think any women acquire cervical cancer?	Strongly agree	254	63
	Agree	109	27
	Neutral	24	6
	Disagree	12	3
	Strongly disagree	4	1
Do you think carcinoma of the cervix can be treated?	Strongly agree	35	8.7
	Agree	75	18.6
	Neutral	94	23.3
	Disagree	171	42.4
	Strongly disagree	28	6.9
Do you think screening helps in prevention of cervical cancer?	Strongly agree	186	46.2
	Agree	162	40.2
	Neutral	43	10.7
	Disagree	9	2.2
	Strongly disagree	3	0.74
Willingness for screening	Strongly agree	100	24.8
	Agree	185	45.9
	Neutral	70	17.4
	Disagree	39	9.7
	Strongly disagree	9	2.2
**Compute attitude towards Cervical Cancer**	Favorable	273	67.7
	Unfavorable	130	32.3

### Practices towards cervical cancer screening

Most of the respondents 351(87.1%), didn’t have any sexual experience. Among respondents who had sexual experience, 36(69.2%) had sexual intercourse at the age of ≥18 years; whereas the smallest proportion of them 16(30.7%) had a history of sexual intercourse at the age of < 18 years. Of all the respondents, only 2 (0.5%) of them had been screened for cervical cancer at least once. With regard to the reasons why participants were not being screened, 211 (52.4%) and 114 (28.3%) of the respondents indicated that they never had experienced the illness and lack of information on the screening place. Among the total respondents, only 5(1.2%) of the respondents were vaccinated (Table 3).

**Table 3:** Practices towards cervical cancer screening among undergraduate female students at the University of Gondar, Ethiopia, April 2018 (*n* = 403)

Variables	Category	Number	Percent (%)
Sexual experience	Yes	52	12.9
	No	351	87.1
Age at the first sex (*n* = 52)	< 18	16	30.7
	≥18	36	69.2
Number of sexual partners(*n* = 52)	Single	33	63.5
	Multiple	19	36.5
Have you ever screened for cervical cancer	Yes	2	0.5
	No	401	99.5
Reason for not screened	I am healthy	211	52.4
	It may be painful	55	13.6
	I feel shy	23	5.7
	I’m not informed about screening place	114	28.3
Do you receive a cervical cancer vaccine?	Yes	5	1.2
	No	398	98.8
Have you had a Pap smear done before?	Yes	2	0.5
	No	401	99.5
If not had done, why?	No interest	3	0.7
	Partners will not allow	18	4.5
	No time	45	11.2
	Never heard of it	337	83.6

## Discussion

The findings of this study showed that, more than half 59.3% [with 95% CI: (54.3, 64.0)] of the respondents, had good knowledge, which is comparable with other studies done in Hawassa (56.8%) [19] and Nigeria (63.0%) [20]. In this study, 90% of the respondents have heard about cervical cancer from different sources, which is comparable with results reported in Nigeria (88.7%) [20]. However, our finding is higher than a similar study conducted in Nigeria (72%) [21], Hawassa (76.8%) [19], Terkey (78.3) [22] and Wollega (54.4%) [18]. The inconsistency may be due to the time variation. Of those, who had heard about cervical cancer, nearly one-third of the respondents (32.8%), got this information from their teachers,29.8% from mass media and 27% from health workers unlike to findings reported in Uganda, 70.2% of respondents got information from the radio [23]. The result is comparable with the study findings done in Wollega University in which mass-medias such as television and radio (36%), brochures and posters (12.4%), health workers (7.3%) and teachers (0.7%) were mentioned as a sources of information about cervical cancer [18].

This research finding showed that more than half of the respondents (52.4%) knew about at least one possible symptoms of cervical cancer such as vaginal foul-smelling discharge and irregular vaginal bleeding and Post coital bleeding. This finding is lower than other report done in Hawassa (67.9%) [19]. Vaginal foul-smelling discharge and irregular vaginal bleeding is responded by one-fifth (20.8%) and 20.6% of the respondents, respectively. A similar study was also done in Mizan Tepi University; Southern Ethiopia [17], 40.67 and 19.14% of the study participants were mentioned vaginal bleeding and foul-smelling vaginal discharge, respectively.

In the current finding, among respondents who knew about risk factors, a quarter (25.1%) of participants reported that having multiple sexual partners is a risk factor for the disease. Among these risk factors having multiple sexual partners (25.1%), Human papilloma virus (15.6%) and early sexual intercourse (11.9%) were mainly reported risk factors for cervical cancer. This result is similar to the previous study conducted in Hawassa [19] and Nigeria [24]. Among female University students, having several sexual partners was recognized as a major risk factor for cervical cancer in South Africa (62.5%) [25] and Nigeria (28.2%) [20]. On the other hand, avoidance of multiple sexual partners (25.1%), avoidance of early sexual intercourse (16.99%) and HPV vaccination (8.2% were mentioned as preventive measures for cervical cancer which is supported by a study conducted in Wollega [18], Hawassa [19] and Tigray [26]. Nearly one-third (34.7%) of the study participants did not know how can prevents cervical cancer which is higher than a report in South Africa (58.5%) [3]. This discrepancy might be due to the time variation. Nearly three-fourth (73.4%) of the respondents knew that cervical cancer can be treated and 23.3, 22.8 and 22.8% of the respondents mentioned surgery, drug and radiotherapy respectively as treatment option supported by a report in Mizan-Tepi [17].

The attitude of the respondents towards cervical cancer and its screening was also assessed in the study. Higher proportion [67.7% with 95% CI: (63.3, 72.0)] of the respondents had a favorable attitude on cervical cancer and its screening which is higher than a report in Hawassa (55.3% [19]. Recently the Ethiopian government is expanding the information regarding cervical cancer and its screening. Most of the respondents, 89.6% with 95% CI: 86.6, 92.3 agreed that early detection of the disease is important for prevention of cervical cancer. In this study, more than half of (56.8%) the respondents perceived that they can acquire the disease which is a little bit higher (49.28%) than other report done in Mizan-Tepi University, Ethiopia [17]. Less than three-fourths (72.7%) of the respondents thought that cervical carcinoma can be transmitted from person to person. This finding is higher than the result reported in Mizan-Tepi, Ethiopia (39.72%) [17].

Regarding to screening practice, only 0.5% of the respondents had done cervical cancer screening. In approximate to the current study, none of the study participants had undergone cervical cancer screening in Wollega prior to the study [18]. The report is lower than a report conducted in Nigeria (9%) [20] and Tigray (17.2%) [26] .The main reasons mentioned for unscreened were feeling healthy, shy during vaginal examination and lack of information where the screening was given.

Among the total respondents, only 1.2% had vaccinated. The result is lower than a study conducted in Malaysia medical and pharmacy students, 3.6% of the study participants took the HPV vaccine [27]. This evidence showed that special attention should be given to the prevention of the disease through early vaccination. This might be because of the late starting of HPV vaccination in developing countries particularly, Ethiopia.

### Limitation

The results about attitude and practices towards cervical cancer screening were based on self-reports of participants, which may answer due to social desirability that may result in over or under estimates. The other limitation can be this study was not including all University campus students in the study area. It is only descriptive study. Due to lack of sufficient literatures, some comparisons were made with different population.

## Conclusion

Despite the University female students had apparently good knowledge and favorable attitude, their screening practice on cervical cancer was very low. Thus, the health sectors and the gender streaming office of the university need to mobilize students to strengthen the uptake the cervical cancer screening practice.

## Data Availability

The datasets used and/or analyzed during the current study are available from the corresponding author on reasonable request.

## Abbreviations

CI: Confidence Interval
HPV: Human Papilloma Virus
KAP: Knowledge Attitude and Practice
SD: Standard Deviation
SPSS: Statistical Package for Social Science
